# ^18^F-FDG PET/CT discriminates whether rheumatoid arthritis patients are in ultrasound remission or not

**DOI:** 10.1093/rap/rkaf125

**Published:** 2025-10-25

**Authors:** Charline Rinkin, Olivier Malaise, Claire Lamaye, Florane Chauveheid, Caroline Gérard, Laurence Seidel, Michel Malaise, Roland Hustinx, Clio Ribbens

**Affiliations:** Rheumatology Department, University Hospital of Liège, Liège, Belgium; Rheumatology Department, University Hospital of Liège, Liège, Belgium; Nuclear Medicine Department, University Hospital of Liège, Liège, Belgium; Rheumatology Department, University Hospital of Liège, Liège, Belgium; Rheumatology Department, University Hospital of Liège, Liège, Belgium; Biostatistics and Research Method Center (B-STAT), University Hospital of Liège, Liège, Belgium; Rheumatology Department, University Hospital of Liège, Liège, Belgium; Nuclear Medicine Department, University Hospital of Liège, Liège, Belgium; Rheumatology Department, University Hospital of Liège, Liège, Belgium

**Keywords:** rheumatoid arthritis, remission, ^18^F-FDG PET/CT, ultrasonography

## Abstract

**Objectives:**

^18^F-FDG PET/CT is used for cancer evaluation but can also quantify joint inflammation in RA. We evaluated its ability to identify patients in remission as defined by US.

**Methods:**

Sixty RA patients underwent clinical, PET/CT and US evaluations (hands and wrists). For PET/CT, the maximum standardized uptake value (SUV_max_) was determined. At the patient level, the highest SUV_max_, number of PET-positive joints and cumulative SUV_max_ were recorded. US evaluation included gray-scale and power Doppler.

**Results:**

At the joint level, SUV_max_ was significantly lower when the joint was in US remission (*P* < 0.0001). Receiver operating characteristics curves identified a threshold of 0.86 to define joint remission with a high positive predictive value of 95.2%. At the patient level, the highest SUV_max_ and cumulative SUV were significantly lower in patients in US remission (respectively *P* = 0.0023 and *P* = 0.045). The highest SUV_max_ threshold of 2.36 demonstrated a high negative predictive value of 92.9% for determining US remission. For the cumulative SUV_max_ threshold, the negative and positive predictive values for determining US remission were both 73%. In contrast, these PET/CT parameters did not discriminate if a patient is in clinical remission or not.

**Conclusions:**

PET/CT can identify RA patients in remission as defined by US. We show that the highest SUV_max_ and cumulative SUV are the two discriminative PET/CT parameters able to determine whether a patient is in US remission. This provides an additional advantage of PET/CT as an objective tool to evaluate RA.

Key message

- PET/CT can identify RA patients in remission as defined by US.

## Introduction

Remission is the ultimate goal of the treat-to-target strategy in RA and is associated with less radiographic progression [1]. However, even when in clinical remission or in low disease activity, some patients can still exhibit radiographic progression [2]. Accurate assessment of joint inflammation with imaging modalities is therefore necessary in addition to clinical assessment. Ultrasonography is the imaging tool most widely used to thoroughly assess joint inflammation and thereby define a strict RA remission. US can detect subclinical inflammation in patients in clinical remission [3], US remission has a high specificity for predicting structural damage [4] and power Doppler (PD) positivity is a risk factor for flare, even if patients are in clinical remission [5]. However, US evaluation is time-consuming and can only be performed by trained physicians.

Today, ^18^F-fluoro-2-deoxy-D-glucose (^18^F-FDG) PET/CT is widely used for cancer evaluation and systemic inflammation workup and RA patients can undergo PET-CT either for differential diagnosis in case of atypical clinical presentation or for staging if a cancer is suspected. However, PET/CT can also be a tool to quantify joint inflammation in RA [6]. Previous studies have demonstrated that PET/CT parameters are correlated to clinical parameters of disease activity and can monitor the therapeutic response in RA [7]. Clinical activity can also be established with PET/CT in other inflammatory diseases with different uptake patterns than in RA [8, 9], suggesting that PET/CT could also differentiate different types of arthritis. Despite this enthusiasm for PET/CT use in RA, relevant data about PET/CT and US parameters are lacking. We previously demonstrated that two ^18^F-FDG PET/CT metabolic parameters [the number of PET-positive joints and the cumulative maximum standardized uptake value (SUV_max_)] were correlated with US parameters in RA [10, 11], while Park *et al.* [12] published significant positive correlations between summed joint SUV with gray-scale US (GSUS) and power Doppler US (PDUS) with ^18^F-sodium fluoride PET/CT. However, no data are available about the ability of PET/CT to determine remission as defined by US. In this study, we evaluated the ability of PET/CT to identify RA patients in remission, as defined by US, both at the ‘joint’ and at the ‘patient’ level.

## Methods

### Study design and patients

Sixty adult patients with RA fulfilling the ACR/EULAR 2010 criteria [13] were recruited at the Rheumatology Department of the University Hospital of Liege between October 2018 and April 2021. The study was approved by the ethics committee of the hospital (B70720108722) and written informed consent was obtained from each patient. The study consisted of assessment of disease activity by clinical examination, composite disease activity scores, US of the hands and wrists and ^18^F-FDG PET/CT. Joint assessments were performed on the same day by three independent investigators (one for the clinical examination, one for US and one for PET/CT), blinded for other outcomes. Clinical examination included the number of tender and swollen joints. Subjective assessments included the patient global assessment on a visual analogue scale (VAS; 0–100 mm) and the HAQ [14]. Blood samples were obtained for evaluation of RF, ACPAs, CRP and ESR. Disease activity was evaluated using 28-joint DAS with CRP (DAS28-CRP) [15] Simplified Disease Activity Index (SDAI) [16] and Clinical Disease Activity Index (CDAI) [17]. Remission cut-off levels were those used in the literature (DAS28-CRP and DAS28-ESR remission if ≤2.6, CDAI remission if ≤2.8, SDAI remission if ≤3.3).

### Ultrasonography

Each US was performed by one experienced examiner using a 10–14 MHz B-mode multifrequency transducer (Logiq E9, GE Healthcare, Milwaukee, WI, USA). US was carried out as previously described [18]. In brief, GSUS and PDUS were carried out on 22 joints for each patient (wrists: radiocarpal and intercarpal, MCP joints 1–5 and PIP joints 1–5). Patient and probe positioning were as recommended by EULAR guidelines [19]. Synovitis was classified according to the OMERACT [20] (definition, measurement of grade from 0 to 3 in GS and in PD). Presence of synovitis, synovitis grade, presence of PD and PD grade were collected at the joint level. The number of joints with synovitis, sum of the grade of the 22 joints (cumulative synovitis grade in GS), mean synovitis grade, number of PD-positive joints, cumulative PD grade and tenosynovitis of the wrists and hands were collected at the patient level. We defined US remission as in our previous work [18]: at the joint level, GSUS synovitis ≤1 and PDUS synovitis 0; at the patient level, GSUS synovitis ≤1, PDUS synovitis 0 and GSUS/PDUS tenosynovitis 0.

### 18F-FDG PET/CT imaging

The PET/CT studies were performed using a Gemini BigBore scanner (Philips Medical Systems, Cleveland, OH, USA). The detailed protocol is described in our previous article [11]. In brief, the patients fasted for 4 h and were injected with ^18^F-FDG. The uptake time was 60 min and the image acquisition sequence was as follows: first, a scout view CT, followed by a PET emission study that included the hands and wrists. Finally, a low-dose CT (5-mm slice thickness) was performed over these joints. The hands and wrists were positioned and held down on a dedicated Plexiglas device to avoid movements between the PET and CT acquisitions. The images were visually analysed and joints were considered positive for synovitis when the ^18^F-FDG uptake was increased compared with the background in areas corresponding to joint synovium on the CT, i.e. either when thickened synovium was recognized on CT or in locations corresponding anatomically to synovium, excluding uptake in other structures such as muscle and tendons. The ^18^F-FDG uptake was then quantified using the maximum standardized uptake value (SUV_max_). In PET-positive joints according to the visual analysis, the SUV_max_ was obtained by drawing a region of interest (ROI) over the most active synovial area identified. When no synovitis was identified, ROIs were placed in the corresponding areas on the CT and drawn around the appropriate joint. The SUV_max_ was determined for each of the 22 joints evaluated: wrists, MCP and PIP joints of both hands. At the patient level, we recorded the number of PET-positive joints, the sum of all SUV_max_ of the 22 joints (cumulative SUV_max_) and the highest SUV_max_.

### Statistical analysis

Results are presented as mean (s.d.) or median (quartiles) for continuous variables and as frequency tables for qualitative variables. Analysis of variance-1 was used to compare means between groups. Logistic regression was used to analyse the US remission with respect to SUV_max_ and odds ratios (ORs) were reported. Receiver operating characteristics (ROC) curves were calculated and the Youden’s method was used to find an ^18^F-FDG PET/CT threshold that can discriminate patients in US remission, or not, at the joint and at the patient level. Sensitivity, specificity, positive predictive value (PPV), negative predictive value (NPV) and their 95% CIs were calculated. A Fisher’s exact test with a Bonferroni correction was used to compare the PET/CT and US remission status of the joints to the clinical evaluation. The results are considered significant at the 5% level of uncertainty (*P* < 0.05). Calculations were performed using SAS version 9.4 (SAS Institute, Cary, NC, USA) and figures with R version 4.4.1 (R Foundation for Statistical Computing, Vienna, Austria).

## Results

### Patient characteristics and disease activity

Sixty RA patients were included and underwent clinical, US and ^18^F-FDG PET/CT evaluation. Detailed clinical, US and PET/CT disease evaluation at the patient level are described in Table 1. Forty-three (71.7%) patients were female, with a median age of 62.2 years (range 26.6–80.6) and a median disease duration of 6.5 years (range 0–30). RF and ACPA were positive in 68.3% and 70.0%, respectively. Forty (66.7%) subjects were taking conventional DMARDs, 31 (51.7%) biologic DMARDs and 26 (43.3%) daily oral prednisolone [mean dose 2.1 mg (s.d. 2.7)].

**Table 1.** Clinical, US and PET/CT characteristics of the RA patient population

Variable (per patient)	*N*	Values	Variable (per patient)	*N*	Values
Patients’ characteristics					
Female, *n* (%)	60	43 (72)	Conventional DMARDs, *n* (%)	60	40 (67)
Age (years), median (min–max)	60	62 (27–81)	Biologic DMARDs, *n* (%)	60	31 (52)
Disease duration (years) median (min–max)	60	6.5 (0–30)	Glucocorticoids, *n* (%)	60	26 (43)
RF, *n* (%)	60	41 (68)	NSAIDs, *n* (%)	60	13 (22)
ACPA, *n* (%)	60	42 (70)	Body mass index (kg/m^2^), median (min–max)	60	27 (17–41)
Subjective evaluation of disease activity			Active smoking, *n* (%)	60	18 (30)
Patient global assessment (VAS mm), mean (s.d.)	60	48.4 (28.0)	Ultrasound characteristics		
Physician global assessment (VAS mm), mean (s.d.)	60	22.6 (24.5)	Joints with synovitis, mean (s.d.)	60	3.12 (4.58)
HAQ (/60), mean (s.d.)	60	16.6 (11.9)	Cumulative synovitis grade, mean (s.d.)	60	5.38 (9.49)
Blood inflammatory parameters			Synovitis grade, mean (s.d.)	60	0.25 (0.43)
CRP (mg/l), mean (s.d.)	60	7.03 (15.9)	Cumulative synovial thickness (mm), mean (s.d.)	60	6.95 (11.6)
ESR (mm/h), mean (s.d.)	58	17.2 (16.2)	Synovial thickness (mm), mean (s.d.)	60	0.32 (0.53)
Clinical examination			PD-positive joints, mean (s.d.)	60	0.22 (0.67)
Swollen joints/patient (*n*), mean (s.d.)	60	3.17 (4.47)	Cumulative PD grade, mean (s.d.)	60	0.40 (1.50)
Tender joints/patient (*n*), mean (s.d.)	60	5.72 (6.34)	PD grade, mean (s.d.)	60	0.018 (0.068)
Disease activity index			Tenosynovitis, *n* (%)	60	
DAS28-CRP, mean (s.d.)	60	3.59 (1.39)	No		47 (78)
Remission, *n* (%)		14 (23)	Yes		13 (22)
Low activity, *n* (%)		12 (20)	US remission, *n* (%)	60	
Moderate activity, *n* (%)		26 (43)	No		28 (47)
High activity, *n* (%)		8 (13)	Yes		32 (53)
CDAI, mean (s.d.)	60	16.0 (13.9)	PET/CT characteristics		
Remission, *n* (%)		9 (15)	PET-positive joints, mean (s.d.)	60	2.47 (3.86)
Low activity, *n* (%)		18 (30)	Highest SUV_max_, mean (s.d.)	60	1.51 (1.80)
Moderate activity, *n* (%)		15 (25)	Cumulative SUV_max_, mean (s.d.)	60	5.60 (9.97)
High activity, *n* (%)		18 (30)			
SDAI, mean (s.d.)	60	16.7 (14.1)			
Remission, *n* (%)		10 (17)			
Low activity, *n* (%)		19 (32)			
Moderate activity, *n* (%)		18 (30)			
High activity, *n* (%)		13 (22)			

At the patient level, there were 3.2 (s.d. 4.5) swollen joints and 5.7 (s.d. 6.3) tender joints per patient. The mean DAS28-CRP was 3.6 (s.d. 1.4). For US evaluation, 53.3% of patients were in US remission, i.e. GSUS synovitis ≤1, PDUS synovitis 0 and GSUS/PDUS tenosynovitis 0. For PET evaluation, there were 2.5 (s.d. 3.9) PET-positive joints per patient. The mean highest SUV_max_ was 1.5 (s.d. 1.8) and the mean cumulative SUV_max_ was 5.6 (s.d. 10.0).

At the joint level, 10% (137/1320) of joints were ‘only tender’, 3% (43/1320) were ‘only swollen’, 14% (180/1320) were ‘tender or swollen’ and 10% (131/1320) were ‘tender and swollen’ at clinical evaluation. US data were available for 1318/1320 joints. Among them, 1219/1318 (92%) were in remission, with remission defined as GSUS synovitis ≤1 and PDUS synovitis 0. When evaluating each subtype of joint separately, remission was observed in 86% of wrists, 92% of MCP joints and 94% of PIP joints. For the PET/CT results, the mean SUV_max_ for ‘any joint’ was 0.25 (s.d. 0.79). If we separately considered wrists, MCP joints and PIP joints, the mean SUV_max_ was 0.85 (s.d. 1.49), 0.19 (s.d. 0.67) and 0.20 (s.d. 0.63), respectively. The mean SUV_max_ was significantly higher for the wrist than for the other joints (*P* < 0.0001).

### Diagnostic performance of PET/CT to identify US remission at the joint level

At the joint level, the metabolic activity (assessed with the SUV_max_) was significantly lower in the joints in US remission, defined as GSUS synovitis ≤1 and PDUS synovitis 0 [SUV_max_ for ‘any joint’ in US remission (1219/1318): 0.18 (s.d. 0.60); SUV_max_ for ‘any joint’ not in US remission (99/1318): 1.21 (s.d. 1.71, *P* < 0.0001)]. The OR for US remission was 0.411. ROC analysis found a threshold of 0.86 for the SUV_max_ to identify remission (ROC curve at the joint level is presented in Supplementary Fig. S1, available at *Rheumatology Advances in Practice* online), with a high PPV [95.2% (95% CI 93.8, 96.4)], but with a low NPV [28.9% (95% CI 21.7, 36.8)]. Sensitivity and specificity were 91.3% (95% CI 89.6, 92.8) and 43.4% (95% CI 33.5, 53.8), respectively (Table 2). If we separately analyse wrists, MCPs and PIPs, the metabolic activity (SUV_max_) was each time lower in joints in US remission (all *P* < 0.0001), with ORs of 0.418, 0.418 and 0.394, respectively. Joint-specific sensitivity, specificity, PPV and NPV are detailed in Table 2. Sensitivity and PPV were high, with a better NPV for the wrist than for the other joints.

**Table 2.** Diagnostic performance of PET/CT to identify US remission at the joint level and at the patient level

Joint	Sensitivity, % (95% CI)	Specificity, % (95% CI)	PPV, % (95% CI)	NPV, % (95% CI)
US remission at the joint level				
‘Any joint’ SUV_max_ (cut-off 0.86)	91.3 (89.6, 92.8)	43.4 (33.5, 53.8)	95.2 (93.8, 96.4)	28.9 (21.7, 36.8)
Wrist SUV_max_ (cut-off 2.36)	96.1 (90.4, 98.9)	58.8 (32.9, 81.6)	93.4 (86.9, 97.3)	71.4 (41.9, 91.6)
MCP SUV_max_ (cut-off 1.36)	94.4 (92.1, 96.2)	33.3 (20.4, 48.4)	94.2 (91.9, 96.0)	34.0 (20.9, 49.3)
PIP SUV_max_ (cut-off 0.86)	91.3 (88.7, 93.5)	41.2 (24.7, 59.3)	96.3 (94.3, 97.7)	22.2 (12.7, 34.5)
US remission at the patient level				
Highest SUV_max_ (cut-off 2.36)	96.9 (83.8, 99.9)	46.4 (27.5, 66.1)	67.4 (52.0, 80.5)	92.9 (66.1, 99.2)
Cumulative SUV (cut-off 2.34)	78.1 (60.0, 90.7)	67.9 (47.7, 84.1)	73.5 (55.6, 87.1)	73.1 (52.2, 88.4)

US remission at the joint level was defined as GSUS synovitis ≤1 and PDUS synovitis 0. US remission at the patient level was defined as GSUS synovitis ≤1, PDUS synovitis 0 and GSUS/PDUS tenosynovitis 0.

### Diagnostic performance of PET/CT to identify US remission at the patient level

At the patient level, the highest SUV_max_ was significantly lower in patients in remission as defined by US than those not in US remission [mean 0.77 (s.d. 1.06) *vs* 2.35 (s.d. 2.08), *P* = 0.0023]. The cumulative SUV_max_ was also statistically significantly lower in patients in US remission [2.78 (s.d. 6.39) *vs* 8.82 (s.d. 12.25), *P* = 0.045] while there was no significant difference in the number of PET-positive joints [1.53 (s.d. 3.43) *vs* 3.54 (s.d. 4.10), *P* = 0.061]. The ROC analysis identified a highest SUV_max_ threshold of 2.36 (ROC curve at the patient level is presented in Supplementary Fig. S2, available at *Rheumatology Advances in Practice* online), with a high NPV for determining US remission [92.9% (95% CI 66.1, 99.2)] and a lower PPV [67.4% (95% CI 52.0, 80.5)]. For the cumulative SUV threshold, the NPV and PPV for determining US remission were 73.1% and 73.5%, respectively.

### Clinical description of joints according to US and PET/CT status

In order to analyse the low specificity of PET/CT to identify US remission at the joint level, we separated the PET/CT and US remission status of the joints according to their clinical characteristics (Table 3). PET-positive joints, although in US remission (i.e. ‘no PET remission, US remission’) were statistically significantly more often ‘tender and swollen’ than joints in US and PET/CT remission (14.2% *vs* 6.1%, *P* = 0.0040; Table 3). These joints were also numerically more ‘swollen only’ (5.7% *vs* 2.2%), although this difference did not reach statistical significance. They were not different in terms of tenderness only.

**Table 3.** PET/CT and US remission status of the joints according to their clinical evaluation

Variable	Total (*N* = 1318)	No PET remission, no US remission (*N* = 43)	No PET remission, US remission (*N* = 106)	PET remission, no US remission (*N* = 56)	PET remission, US remission (*N* = 1113)
	*n* (%)	*n* (%)	*n* (%)	*n* (%)	*n* (%)
Tender only	137 (10.4)	4 (9.30)	13 (12.26)	8 (14.29)	112 (10.06)
Swollen only	43 (3.3)	7 (16.28)^a^	6 (5.66)	6 (10.71)^a^	24 (2.16)b,c
Tender and swollen	131 (9.9)	22 (51.16)a,d	15 (14.15)a,b,c	26 (46.43)a,d	68 (6.11)b,c,d
Tender or swollen	180 (13.7)	11 (25.58)	19 (17.92)	14 (25.00)	136 (12.22)

a Significantly different from PET remission and US remission.

b Significantly different from no PET remission and no US remission.

c Significantly different from PET remission and no US remission.

d Significantly different from no PET remission and US remission.

Conversely, US-positive joints in PET/CT remission (i.e. ‘PET remission, no US remission’) were not statistically significantly different in terms of swelling and tenderness from joints that were not in remission, neither by PET/CT nor by US. However, they were statistically significantly more often swollen than joints in US and PET/CT remission (*P* = 0.0022 for ‘swollen only’ and *P* < 0.001 for ‘tender and swollen’).

The percentage of ‘only tender’ joints was similar in all four subgroups defined by the status of remission or not according to US and PET/CT. In contrast, the percentages of ‘swollen only’ and ‘tender and swollen’ joints were significantly lower in joints in US and PET/CT remission compared with joints not in remission by US or PET/CT (*P* < 0.0001 for both).

### Diagnostic performance of PET/CT to identify clinical remission at the patient level

Lastly, we wondered if PET/CT could also discriminate patients that were, or not, in clinical remission. There was no significant difference in terms of the highest SUV_max_, cumulative SUV_max_ or number of PET-positive joints between patients who were in clinical remission or not, whatever the clinical score used (*P* = 0.19, 0.16 and 0.14, respectively, for DAS28-CRP; *P* = 0.31, 0.25 and 0.23, respectively, for SDAI; *P* = 0.40, 0.37 and 0.29, respectively, for CDAI).

## Discussion

These data demonstrate for the first time that ^18^F-FDG PET/CT can identify RA patients in remission as defined by US in hands and wrists, which are the most commonly affected joints. At the joint level, unitary SUV_max_ was significantly different if the joint was under US remission or not, whether it was a wrist, MCP or PIP. Using ROC analysis, we determined a cut-off of 0.86 that can be used to differentiate if a joint is in remission or not, with a high PPV (meaning that if the SUV_max_ is <0.86, we can reliably attest that this joint would also be in US-defined remission). The opposite is not necessarily true since the NPV is low. If we differentiate joints by subtype, we found a higher mean SUV_max_ in wrists, with a higher SUV_max_ cut-off to discriminate if the wrist is in remission than for the other joints. The NPV for the wrist is also better than for the other joints. Our observations at the joint level, with a better sensitivity than specificity for remission status, are in accordance with our previous work [10], where we observed that the number of PET-positive joints was higher than the number of US-positive joints, while most of the PET-positive joints were also positive by US. This lack of specificity can in theory be due to PET/CT false positives, with fixation due to conditions other than RA (such as osteoarthritis), but also can be linked to a greater sensitivity of PET/CT to detect residual synovial inflammation.

To address the question of the low specificity of PET/CT to identify US remission, we analysed the clinical characteristics (tender and/or swollen) of the joints according to their US and PET/CT remission status. We observed that the ‘tender’ status did not discriminate joints being or not in US and/or PET/CT remission. In contrast, the ‘swollen’ status at clinical examination was different according to the subgroup. Of interest, joints in US remission, but with PET/CT activity, were statistically significantly more swollen (but not more tender) than joints in US and PET/CT remission. The association with a more swollen status (an objective evaluation) indicates that these joints are probably not just ‘truly false positive’, and that PET/CT could possibly be linked to low-grade residual synovitis, given our definition of US remission (GSUS synovitis ≤1 and PDUS synovitis 0). Further studies, e.g. with MRI, are needed to better understand the origin of this PET/CT fixation in the US/PET discordant joints.

At the patient level, we demonstrated that the cumulative SUV_max_, as well as the highest SUV_max_, were able to discriminate if a patient is in US remission or not. The highest SUV_max_ exhibited a very interesting sensitivity and sensibility to estimate US remission, meaning that a single-joint metabolic value on PET/CT—i.e. the highest SUV_max_ among the 22 joints studied—is able to predict RA remission, as defined with US. These two parameters (cumulative SUV_max_ and highest SUV_max_) are important because they allow the fortuitous use of PET/CT to also analyse the remission status of RA patients. While we previously demonstrated that the number of PET-positive joints was significantly associated with several US parameters [10, 11], it failed to discriminate US remission in the present study. Furthermore, the number of PET-positive joints was associated with US parameters in another study in active RA patients with TNF inhibitor failure, but this association was lost after rituximab treatment [21]. In contrast, the association between cumulative SUV and US parameters was maintained after rituximab treatment [21], again suggesting a superiority of cumulative SUV_max_ over the number of PET-positive joints for the evaluation of RA. In contrast, neither highest SUV_max_, cumulative SUV_max_ nor the number of PET-positive joints was able to discriminate patients in clinical remission or not.

Our study has limitations. The first limitation is the lack of radiographic data, which did not allow us to evaluate if conditions other than RA (such as osteoarthritis) could explain PET/CT false positives. However, radiographic data from our previous study [11] showed that PET-positive US-negative joints mainly exhibited a rheumatoid or normal aspect and not an osteoarthritic X-ray pattern. Furthermore, US evaluation was limited to 22 joints (hands and wrists) and our cut-off did not integrate other joints that can be involved in RA, such as knees and feet. Other limitations include the cross-sectional status of this study, the absence of a second cohort of validation for our SUV cut-off and the technical particularities of each PET/CT that can influence the cut-off. Finally, our study did not address the question of the use of PET/CT in everyday clinical practice to evaluate disease activity in RA patients. PET/CT is irradiating and time-consuming (especially if joint delimitation is needed). Therefore, it does not seem appropriate to use it as a first-line imaging tool in RA metrology. However, PET/CT can be routinely used in some RA patients to evaluate inflammation with no evident aetiology or as an initial workup when a cancer is suspected (especially when considering the use of check-point inhibitors, inducing a risk of RA flare). In these situations, RA disease activity evaluation could be proposed in addition to the initial purpose of the PET/CT. Furthermore, PET/CT has the potential to provide a whole-body evaluation and could perhaps, in the future, be systematically proposed for differential diagnosis, evaluation of multisystemic involvement, cardiovascular complications and lung fibrosis as well as cancer screening with a single imaging exam.

In conclusion, we show that the highest SUV_max_ and cumulative SUV_max_ are two quantitative PET/CT parameters able to identify patients in remission defined by US in the hands and wrists. PET/CT objectively identifies RA patients—and joints—in US remission or not. This provides an additional advantage of PET/CT as an objective tool to evaluate and manage RA.

## Supplementary Material

rkaf125_Supplementary_Data

## Data Availability

Data are available upon reasonable request.
