# Supplementary material for: 18F-FDG PET/CT discriminates whether rheumatoid arthritis patients are in ultrasound remission or not
Source: Rheumatol Adv Pract. 2025 Oct 25;9(4):rkaf125. doi: 10.1093/rap/rkaf125 (PMC12617742; doi:10.1093/rap/rkaf125)

**Supplementary Figure S1. Receiver Operating Characteristic (ROC) curve illustrating the diagnostic performance of SUVmax for distinguishing joints under US remission or not.** AUC: area under the curve; MCP: metacarpophalangeal joints: PIP: proximal interphalangeal joints.

**Alt text:** Figure illustrating the ROC curve about the diagnostic performance of SUVmax for distinguishing joints under US remission or not.


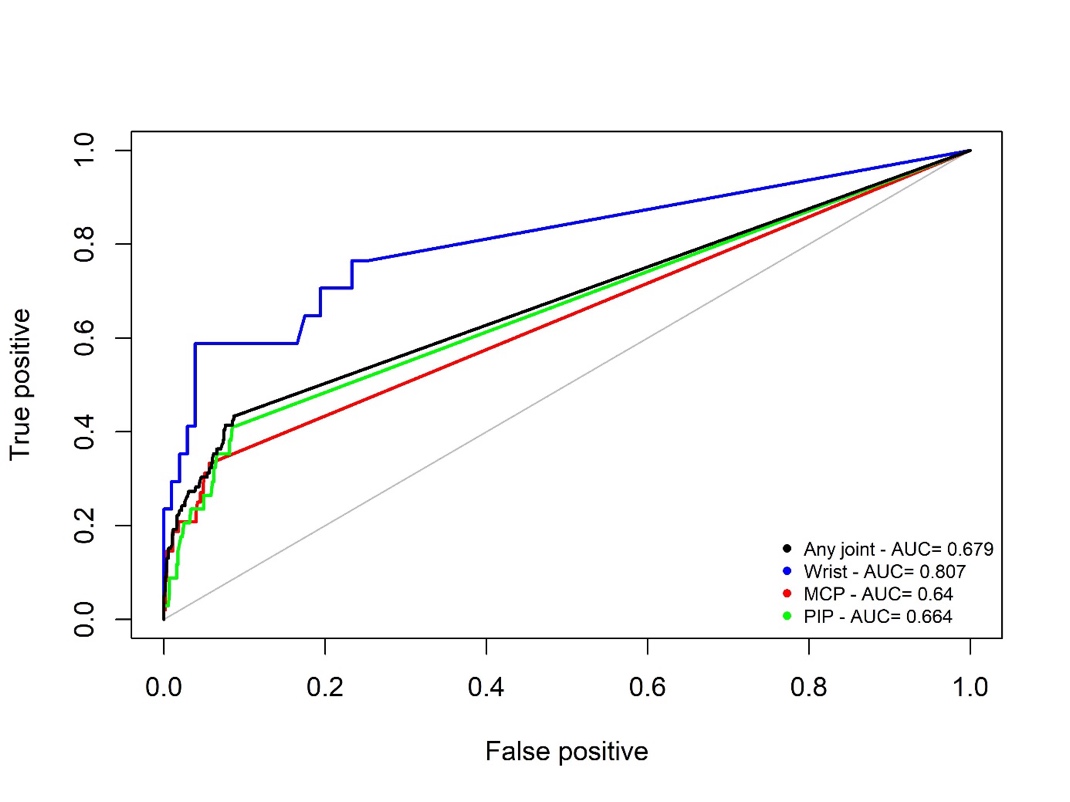


**Supplementary Figure S2a and 2b. Receiver Operating Characteristic (ROC) curve illustrating the diagnostic performance of the highest SUVmax (Figure 2A) and cumulative SUVmax (Figure 2b) for distinguishing patients under US remission or not.** AUC: area under the curve

**Alt text:** Figure illustrating the ROC curve about the diagnostic performance of the highest SUVmax (Figure 2A) and cumulative SUVmax (Figure 2b) for distinguishing patients under US remission or not.


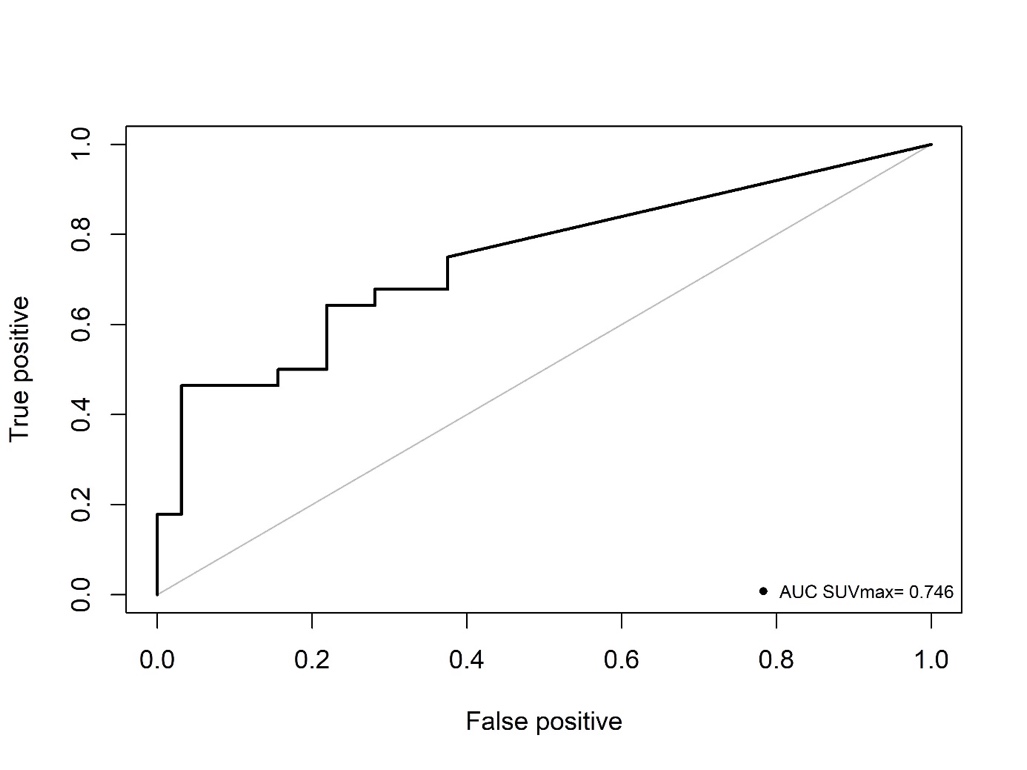


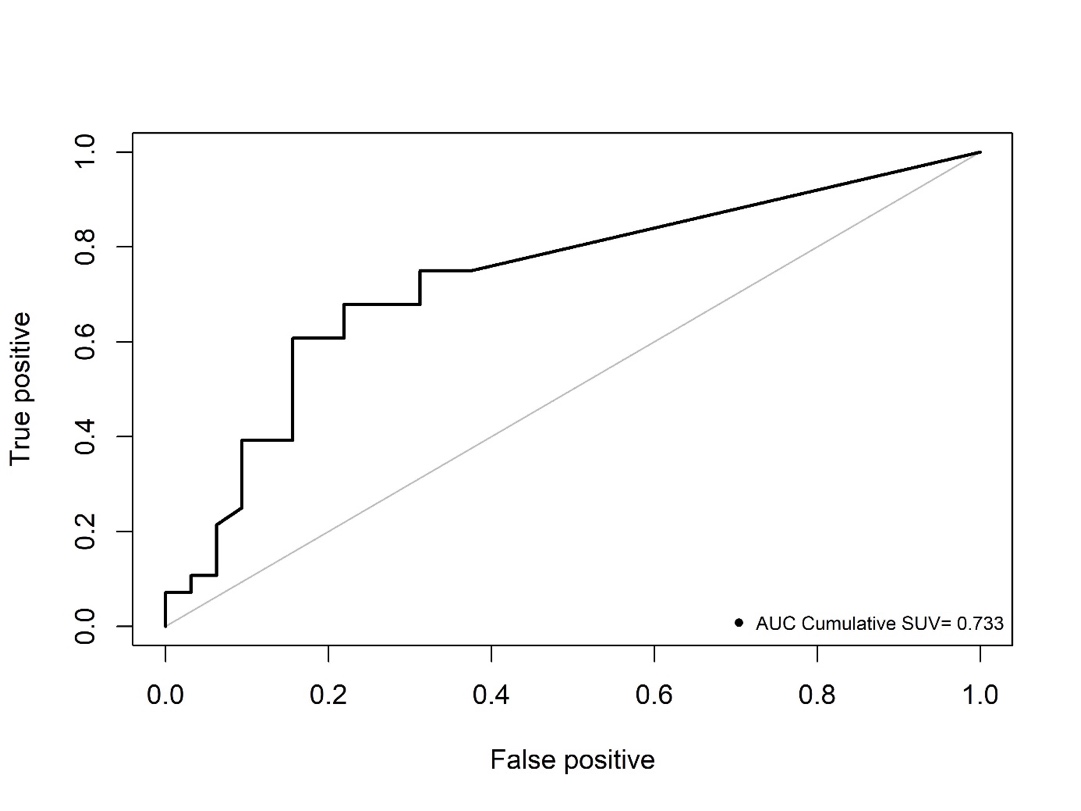

Supplement: rkaf125_Supplementary_Data [file rkaf125_supplementary_data.docx]
